# Analysis of the Impact of Human–Cobot Collaborative Manufacturing Implementation on the Occupational Health and Safety and the Quality Requirements

**DOI:** 10.3390/ijerph18041927

**Published:** 2021-02-17

**Authors:** Alena Pauliková, Zdenka Gyurák Babeľová, Monika Ubárová

**Affiliations:** Institute of Industrial Engineering and Management, Faculty of Materials Science and Technology in Trnava, Slovak University of Technology in Bratislava, 917 01 Trnava, Slovakia; zdenka.babelova@stuba.sk (Z.G.B.); monika.ubarova@stuba.sk (M.U.)

**Keywords:** human–robot cooperation, cobot, employee, industrial enterprises, occupational health and safety, risk prevention, work environment

## Abstract

Implementing Industry 4.0 and interconnected robotization in industrial enterprises drifts towards occupational changes. Nowadays, the task is to create cooperation and collaboration between a robot and a human in a common robotized workplace so that it is safe and effective. The type of robot, the robotic device that works in collaboration with a human operator, is called a cobot. In the case of a closer interaction of the robot or cobot with humans, it is necessary to consider where it is possible to replace human work entirely or where it is possible to merely supplement it. The most socially acceptable option is the implementation of robots only for the performance of supplementary tasks, since the traditional work positions of people in manufacturing processes would remain largely preserved. On the other hand, workplace robotization is particularly suitable for work environments with hazardous chemical substances that are carcinogenic and toxic to humans. Similarly, robotization helps to improve workplace ergonomics and also to avoid, for humans, very laborious and often repetitive work. The SWOT analysis (analysis of Strengths, Weaknesses, Opportunities, and Threats) was used as a relevant tool to assess various aspects of the impact of increasing robotization on working positions in industrial enterprises. SWOT analysis is an indicative assessment of the suitability of implementation of robots in a given workplace, which helps to create an optimal solution and indicate new areas of needed analysis and research directions.

## 1. Introduction and Theoretical Background

The industrial revolution represents a huge development of science and technology. The fourth industrial revolution is known as Industry 4.0. This revolution is realized by a combination of numerous physical and digital technologies such as adaptive robotics, additive manufacturing, artificial intelligence, augmented reality, cloud computing and the Internet of Things. The main purpose of industrial transformation is to increase the competitiveness of companies through resource efficiency and productivity increase [1]. For maintaining and increasing the competitive power of the company, quality and safety are essential in every industry, and have become a matter of survival and must be considered in all management decisions [2]. The quality of production and quality management have a great impact on the performance of industrial enterprises as well as in the agricultural sector [3,4]. In addition to the quality of its products and services, the success of any organization also depends on the performance of the processes taking place within organizations [5]. Industry 4.0 as a technological revolution is characterized by triggering technologies, great automation and computerization [6]. It is therefore important for practical field research to map the readiness of industrial enterprises for the fourth industrial revolution, Industry 4.0 [7].

Significant relevant organizations and experts monitor and analyse the current state of implementation of robots and their safety in the integrated human–robot system [8,9,10,11,12]. The Report from World Robot Summit states that global sales of industrial robots have increased by 114% in the last five years (2013–2017). In the next three years, the market should grow by an average of 14% per year [13]. There are more and more robotics-oriented research programs in Europe, and Europe has a significant share in the production and deployment of industrial robots [14].

An industrial robot is an automatically controlled, reprogrammable, multi-purpose manipulator [15]. Robots play an important role in productivity growth. Their adoption will be a determinant of productivity growth. The economic reasons for adopting robots are stronger in high-wage economies than in low-wage economies [16]. From a long-term perspective, it is more cost-effective to replace human labour with robots in higher-wage economies. It is also more efficient to introduce robots in multi-shift productions, when the robot replaces several employees during the day. It is assumed that robots will work more hours than humans in the future. Since 2000, robots have replaced approximately 1.7 million jobs, including 400,000 in Europe, 260,000 in the United States and 550,000 in China, according to analysts at Oxford Economics (London, United Kingdom). By 2030, robots could replace up to 20 million jobs worldwide. Oxford Economics forecast that each new industrial robot would eliminate 1.6 production jobs, with repetitive work being most at risk [17].

The likelihood of automating and robotization of repetitive work affects not only the future of occupations [18] but can also widen the gender pay gap, especially in areas where there are more significant differences between the pay of men and women [19]. This applies in particular to professions where women are employed mainly in professions, where they perform simpler manual work. The utilization of robotics does not necessarily lead only to technological unemployment. As some professionals conclude, it is likely that part of the technological unemployment can be well compensated by reducing the number of hours worked per year [20]. Thus, there may be two aspects to the impact of robotics on employment. On the one hand, they can replace some professions, on the other hand, they can reduce an employee’s workload by performing some work tasks with a robot.

Robots seem to threaten employment in the short term but will create many new jobs in the long term. Above all, it relieves people from tedious routine activities, as mechanical machines once liberated employees from hard work. Robots will thus work “shoulder-to-shoulder” with humans, which is why the concept of cobots (collaborative robots) has been developed as assistance to manufacturing workers in demanding operations. The idea of designing a robot that would work directly with a human was born back in 1995 as part of a research project by the General Motors Foundation [21]. These types of robots are therefore relatively new. Thus, the standards related to human safety are only in the process of being developed.

Cobot is a type of robot, a robotic device that works in collaboration with a human operator. Cobot provides assistance to the human operator [22]. Unlike conventional industrial robots, which are usually isolated from workers to avoid physical contact with humans, collaborative robots, known as cobot coexist with humans in a common workspace and work with them to perform required tasks [23,24]. Cobots contain safety devices and are considered to be cooperating robots that can work safely with human workers [25,26]. It is also the designation of robots as cooperating and co-existent that may lead to being associated with safety [27]. Although these robots are equipped with safety systems, it is impossible to claim that they will always be safe. As a result, it is necessary to always carry out a thorough risk analysis and safety assessment of a robotized workplace with respect to humans. For this reason, we are assessing the implementation of robotization in industrial companies based on a forecast of new job positions. During the interaction of the robot, or cobot, as they are called, with a human, it is necessary to consider carefully where it is possible to completely replace human work and where it is possible to merely supplement it. Nowadays, robots are a common part of manufacturing operations. The least threatening to workforces would be the implementation of robots in production processes for the purpose of supplementary task fulfilment only, as the traditional jobs performed by people would remain largely preserved. However, such a solution is challenged by the economic pressure, which rather promotes a substitutional approach in which robotization replaces the work of individuals or groups of employees. Overall, less staff will be needed for routine work and specific tasks.

The importance of determining safety in a collaborative environment could also be linked not only to what activities will humans and robots provide side by side but also to the skill level of the person supervising or operating with the robot [10]. This transformation will lead to a situation where the industry will need fewer less specialized workers performing predominantly manual tasks and will, on the other hand, demonstrate a high demand for university-educated workers with skills related to robot interaction [28]. The biggest challenge today is to increase the level of occupational digital literacy. This can begin by evoking curiosity and interest in artificial intelligence and robotics, in an effort to make people better understand these sophisticated modern technologies. Permanent change of the world towards a digitalized “empire” also changes jobs. Therefore, it is necessary to let artificial intelligence and robots do things they know and can do and allow people time to pursue innovations—for example, inventing new products. This new task will bring new advantages to human employees [29]. Consequently, attention needs to be paid to the required competencies of employees [30].

The role of employers in the context of information technologies and cobotic revolution is to work out the balance between jobs, i.e., employment, purposeful replacement of job positions and overall robotization of industrial production. Almost every job posting in an industrial setting includes a requirement that the employee should have a creative approach. So far, the robots are missing this requirement. They lack the imagination and ability to invent new, better practices. Therefore, it is advisable to look at the robot as a real co-worker who can minimize the risk of work-related injuries.

The aim of the presented research was to identify, in addition to the positive effects, the negative effects on humans in human–cobot interaction (HCI) while ensuring the requirements for health and safety at work and compliance with quality requirements in the production process. The purpose of the research was to provide an overview of the possible negative effects of HCI, and on the basis of this identification to enable to propose preventive and corrective measures.

## 2. Risk Prevention Support for the Cobot Implementation in Industrial Manufacturing

The use of robots for manufacturing processes cannot eliminate the risks to zero. The interaction with robots can expose an employee to health and safety risks related to automated machines associated with psychosocial stress [6]. Therefore, adjustment, inspection, or preventive and predictive maintenance will still be necessary. The question of safety during human–robot interaction is very important. Thus, it is needed to implement and test new security features and to provide cobots intrinsically compliant to maintain safety [24]. The nature of the risks during the manufacturing process shifts according to specific stages of the production or handling process. This will mainly involve configuring robots and their maintenance, while performing a number of manual work tasks with the possibility of injury. Safety studies show that many accidents at a robotized workplace occur during non-routine operating situations such as programming, maintenance, testing, setup or customization. During these operations, the employee may temporarily access the robot’s workspace, where unintentional operations may cause injury to a person. Other accidents involving a robotic work environment depending on the entire set of inputs and outputs that the environment receives and releases [31]. Measures to reduce occupational health and safety risks must be part of the robotized workplace project. Robots are an indispensable tool in dealing with exposure to dangerous jobs. This mainly involves working with chemical, carcinogenic, and toxic substances.

During the risk analysis process, it is essential to carefully consider the zones for a human-operator and robot in the robotic workplace so that all risks for humans are eliminated. At the same time, it is important to ensure ergonomic requirements regarding the placement of control panels, safety barriers and locking systems. Robots significantly improve the overall ergonomics of workplaces as well as avoid strenuous and repetitive work operations. Substituting robots in hazardous operations and reducing stress from work with heavy loads reduces the emergence of occupational illnesses, accidents and injuries. The basis for ergonomic solutions is provided by Decree of the Ministry of Health of the Slovak Republic No. 542/2007 in Annex 1 *Requirements for the place of work in connection with the reduction of increased physical stress at work.* Table 1 provides an overview of controls-types, shapes, positions, method of control and control forces [32].

Robots dispone with a variety of sensors that are used to identify and make inferences about their environments and their state. These sensors often are not only noisy but leading to uncertainty in the state of the robot which can cause errors while performing. This is the reason for human supervisors to be often required monitoring of robot execution and reduce the uncertainty of robot performance [33].

Nowadays, automated systems cannot work without human intervention. That is why, even at this level of technology, it is necessary to respect human beings and to create suitable safe working conditions in terms of ergonomics and physical load, e.g., unilaterally strenuous movement. The maximum torso rotation angles while performing a task, torso bending and working height are dimensions that need to be required for installing buttons, levers and switches. The torso rotation is at a maximum angle of 45° to both sides and a max of 60° for turning the head during less frequent activities. In a robotic workplace or during the configuration process and maintenance, the less frequent activities have so-called acceptable 60° for bending. These ergonomic requirements must also be observed when designing robotic equipment. On the other hand, the robot/cobot has no physiologically limiting angles of rotation and bending. In those cases, if a human cannot perform the task, the robot can [31].

Although the technical challenges associated with the design, implementation and deployment of human–robot collaboration systems have been overcome, the safety of operators will always be a major factor for acceptance achievement. To ensure operator safety, existing applications mainly separate humans from robot workspaces [34].

As is argued in some studies, well-managed safety and health at work require properly managed risks. If the potential risks and the assessed risks are not correctly identified, they cannot be adequately managed. It often happens that the risk assessment process is only a formal matter. External health and safety services are limited by the price offer [35].

Some studies point to a lack of information and the ambiguity, inconsistency of reactions when people move and place movements at different heights [36]. Since workers, unlike robots, are not unified, it is also necessary to take into account the physical differences between individual workers. Moreover, the interaction with people is also the challenge for the research the system architecture for launching the action based on the instruction to perform the assigned task [37]. Unknown objects for the robot can also be a challenge for technological solutions [38]. Better results for navigating robots have been reported if a human worker is not tagged as an obstacle [9].

The risk prevention support during the implementation of the cobot within the industrial operations is twofold. Applicable ISO standards are voluntary and guide the implementation process while minimizing errors and waste. On the other hand, legislation is mandatory and must be followed as written. Safety regulations in a robotized workplace are defined with aim of eliminating hazards by designing suitable equipment, applying safety equipment (e.g., safety buttons or sensors), providing safety and health markings (e.g., warning signs), educating and training employees in production, programmers and service personnel (training and practical exercises) and by using personal protective devices [31]. When talking about the safety of the robotic workplace itself, the legislative obligations are shared between the manufacturer and the operator. The manufacturer and his team (designer, developer, etc.) build a robotic device in accordance with ISO 12100: 2011 and ISO 10218-1: 2011 and, based on the risk assessment, the team designs the workplace configuration [15,39]. The technological level or technological maturity of robots’ applications is different. The ways in which robots move are characterized by a wide range of patterns. Some of them have been used for decades and can be easily controlled by non-professional users. Others have had little success, but their operation is well known and documented [40]. The configuration of a robotized workplace is a complex task. The manufacturer of a cobot issues a declaration of compliance pursuant to Act No. 56/2018 Coll. on Conformity assessment of a product, making a designated product available on the market and amending certain laws. Within the Act, Paragraph 4 defines the *Intended product*, and Paragraph 5 of the same Act defines the obligations of a producer. Further, Paragraph 6 outlines the duties of an authorized representative, and Paragraph 7 continues with the obligations of an importer, before placing the designated product on the market. The designing team selects how to perform the compliance assessment according to Paragraph 22. This paragraph defines basic procedures for conducting the assessment [41].

After the completion of the configuration of the robotized workplace comes its implementation and integration into the operation process. A newly designed, standalone robotized workplace is significantly different from a workplace that is being integrated into an already existing system. Furthermore, of course, there is a difference whether this is an application with an industrial robot working alone or a collaborative, human–robot, application. The operator proceeds in accordance with Act no. 124/2006 Coll. on *Occupational Health and Safety and on Amendments to certain Acts* and Decree of the Government no. 392/2006 Coll. *Regulation of the Government of the Slovak Republic on Minimum health and safety requirements for the use of work equipment* [42,43].

In standard ISO 10218-2: 2011, Annex A lists hazards that may be associated with the robot. The manufacturer is required to carry out a hazard analysis, i.e., assess the risks and eliminate or reduce them to an acceptable level using an appropriate design. In identifying hazards, consideration shall be given to at least the following areas:

- The characteristics of the robot, its speed, force, geometric shapes, material, etc.,
- Anticipated positions of the operator with respect to robot’s proximity, and anticipated contact points of the operator with the robot;
- Operator’s movement,

Risks resulting from a shape or surface related characteristics of workpieces, i.e., sharp edges, possible protrusions, slippery surface, etc. [44].

In addition to Annex A, listing significant hazards, standard ISO 10218-2: 2011 contains Annex C specifying safeguarding material entry and exit points and Annex G specifying means of verification of the safety requirements and measures [45].

Requirements for the implementations of robots and cobots in the workplace are formulated mainly in the standards ISO 10218-a: 2011 and ISO 10218-2: 2011, which are valid at the international level. These standards were developed based on several standards listed in the references of these standards. Sufficient legislative requirements specifying robot safe performance or human–robot or cobot interaction at the national level are still absent.

## 3. Methods and Results

Based on the above-mentioned relevant standards and legislation in force, we modelled a SWOT analysis whose main objective, in our case, was to assess the requirements for the robotization of production processes in relation to occupational health and safety (OHS). The need to assess the requirements for the implementation of cobots arose from the interest of management in industrial enterprises, which planned to introduce them as part of the expansion of the industrial park. Experts from the manufacturing enterprises formed a working group, which consisted of managers and technologists from the parent organization and twenty-three subsidiaries with the participation of the authors of the paper as consultants. At repeated meetings, they identified criteria that were subsequently organized into 4 dimensions depending on their external and internal origin and positive or negative impact, respecting existing standards and applicable legislation.

The matrix shown in Table 2 summarizes the individual criteria, which are organized into 4 dimensions of external and internal origin. It is necessary to continuously develop strengths and strive to eliminate weaknesses, which is a prerequisite for minimizing risks and maximizing the opportunities offered. It is equally important to concentrate efforts on turning weaknesses into strengths. Furthermore, in some cases strengths can create weaknesses. It is also necessary to consider the weight of an individual criterion.

Subsequently, after criteria identification, it was necessary to transform the criteria into quantifiable values in order to make them measurable. The quantification of the criteria was important to enable managers to implement decisions based on concrete values. Saaty’s method [45,46,47] was also used to assess the correlation between individual strengths and weaknesses on one side and the decisive changes (opportunities and threats) in the external environment on the other side. Table 3 provides the point scale with the descriptors. Table 4 indicates the significance of the individual criteria. Due to the reducing nature of weaknesses and threats on the implementation of robotization we used a value of (−1) as a multiplier.

These point and value tables (Table 3 and Table 4) served, together with benchmarking and brainstorming, as a base for determining the values listed in Table 5 (Saaty’s matrix and IFAS matrix—for internal factors analysis) and Table 6 (Saaty’s matrix and EFAS matrix—for external factors analysis). Experts in the working group evaluated the proposed criteria, which were scored and the EFAS and IFAS matrices were created.

A pairwise comparison in Table 5 indicates that the most significant benefits of introducing robotics are the elimination of dangerous activities in the work environment, and the reduction of the risk of occupational illnesses, injuries and accidents. The most significant weakness is the possibility of occurrence safety risks in a collaborative workplace. This analysis makes evident that this weakness significantly reduces the strengths in the implementation of robotization in the industrial enterprise and it needs to be properly addressed. The value of the difference between strengths (S) and weaknesses (W) is 2.25.

Pairing comparison in Table 6 indicates that the most important opportunity in introducing robotics is to increase the competitiveness of industrial operations and the opportunity to appropriately combine human and robot/cobot skills in a collaborative workplace. The most significant threat is a lack of qualified professional, a trend that is already evident, especially in the automotive industry [48,49,50]. The value of difference between opportunities (O) and threats (T) is 2.

Based on results shown in Table 5 and Table 6, values were calculated for a complete SWOT analysis, used by managers to select the appropriate strategy. The SWOT analysis is a synthesis of the results of the analysis of Saaty’s, IFAS and EFAS matrices. Its basic contribution lies in the ability to determine the strength of individual impacts on the formulation of the organizational strategy in the implementation of robotization with regard to occupational safety. The result of the combination of these matrices and their analysis is a quantitative determination of the value, which is an indicator for the selection of a suitable variant of the strategy of robotics implementation.

Table 7 indicates a discreet SWOT analysis that individually evaluates weaknesses, strengths, opportunities and threats. The result of the analysis shows that the ST strategy is the most valuable one with a value of 8.47. If the MAX–MIN confrontation strategy is applied, it means that strengths will be maximized and threats suppressed. In the field of robotics implementation, this strategy means that the focus on the robotic workplace is always with respect to the human being, i.e., the elimination of activities that create danger in the working environment and other burdens related to the ergonomic requirements of the workplace. The lack of qualified professionals can be mitigated by proper training, transfer from other operations or contracting them from abroad.

As some authors have argued, the combination of the advantages of direct cooperation between humans and robots is interesting for industrial enterprises. While the necessary technologies are already available, there is a lack of relevant safety standards to occupational ensure safety and are one of the main barriers in establishing direct cooperation between humans and robots [8].

## 4. Discussion

The development of autonomous, cooperating industrial robots belongs to the technological priorities of the development of the intelligent industry of the Slovak Republic [51,52]. Some authors strengthened the economic viability of the implemented manufacturing and robotization of production processes. According to their recalculations, significant savings are recorded in the budgets [53]. Robotization in the industry is not just causing changes in the structure of employment. Robotization allows to increase the repeatability and quality of production, allows to obtain stable production parameters, high accuracy, and thus allows the production of quality products and in addition, the increase in productivity [54]. Regarding productivity, robotized enterprises are more efficient than non-robotized enterprises. What is more, employees are more intensively trained and rewarded at robotic enterprises compared to non robotized enterprises [55].

In terms of occupational health and safety, the expansion of technologies with the potential robotization brings not only opportunities, but also challenges. One of the greatest benefits (see SWOT analysis in Table 5) is the opportunity to replace people working in harmful or hazardous environments. In the field of defence, security or nuclear industry, but also in logistics, maintenance and inspection, autonomous robots are particularly beneficial to replace workers who perform unclean, monotonous or dangerous tasks, thus preventing them from being exposed to risk factors and settings, while reducing physical and ergonomic risks [56].

Robots can be and are already being used to perform repetitive and monotonous tasks, i.e., welding centres. This appears to be the greatest benefit of automatization of otherwise routine work. In the future, many other, often recurring, high-risk or unpleasant tasks, will be performed by robots in a variety of sectors such as agriculture, construction, transport or healthcare. However, despite the rapid advancement in robotics, people will still be better suited to perform certain tasks that robots are not proficient at, e.g., reaction time or dealing with unpredictable situations.

Improving the flexibility and reliability of cobots, as well as the issue of cobot autonomy, remains a challenge in the collaboration of cobots with humans [57]. Practice and carried out researches pointed that the issue of safety is an important area, which needs to be analysed and evaluated before the solution of mutual cooperation between human and robot is installed in industry. However, there is great potential for collaboration with robots [34].

The primary challenge is to achieve the most suitable balance of abilities between humans and robotic. This was presented in the SWOT analysis as an opportunity. The advantages of robotization include the repetition of monotonous, very precise or strenuous activities. On the other hand, humans are creative, flexible and adaptable to perform both monotonous and strenuous activities. However, humans can do that only for a short period of time. Prolonged exposure to these activities could affect their health and safety. Another challenge is to find an ideal balance in the interaction between robots and people in a common workplace. There will be closer contact between robots and people in the workplace, leading to the development of new approaches to human safety and protection in a human–robot system. Some European countries include robotics in their national programs and seek to promote safe and flexible cooperation between robots and operators [56]. When comparing the work environment with and without the robot, there are significant differences in risks, especially for operators. These are motion predictability, movement speed, and strict division of robot and operator zones. Very important is the definition of common zones while observing safety conditions.

Creating and implementing plans and guidelines for human–robot collaboration imposes also additional challenges on robot supervisors in terms of social rules and legibility. The design and implementation of such plans differ in domains with static or relatively static people and in environments with people who move [9]. The introduction of new technologies to assist workers to achieve effective working performance requires not only a need for new health and safety management requirements for monitoring emerging risks, but also raises new legal and ethical issues. Due to differences in the development of different fields of application, it is not possible to provide uniform risk and safety management guidelines. Individual analysis to identify potential risks and dangerous activities of autonomous robotic technologies needs to be carried out for each specific industry, such as agro-food industry, nursing services, home services, manufacturing, professional services and transport [56]. Given that robots will be used in many industries, we are able to estimate their impact and benefits on today’s monotonous and unilateral tasks and new workplace ergonomics.

Changes in the implementation of work processes tend to cause some types of jobs to disappear and new ones to emerge. We need to prepare people for new positions. We need educated and skilled people who will be able to set up, maintain, and program robotic equipment. The great risk of robotized workplaces is, and will be, the lack of qualified employees. It turns out that automatic and robotic technology will not be able to completely exclude a person from the working process in the near future. Therefore, it will be necessary to continue paying attention to the ergonomic aspects of robotization.

Additionally, the impact of robotics on employees’ motivation and well-being is not yet widely understood. Similarly, we cannot assume emerging psychosocial factors related to robotization. That is why in addition to the various aspects related to and cooperation of the human and robot, it is important to pay attention to the degree of acceptance of cooperation with the robot by the human. So far, studies are known that examine the psychological perception of human–robot interaction [58,59]. As can be observed, the more technical devices we have in life, the more we want them to be socially intelligent [60]. Some studies suggest that humans are more willing to interact with a robot that appears human and is more likely to respond positively [61]. On the other hand, there is also argued that robots need to be understood as tools and parts of property under human control that should be taken into account in their design. Therefore, robots should not be designed in a way that elicits anthropomorphizing reactions and should not replicate the behaviour associated with good colleagues [62]. Ergonomic parameters are also important when designing robots [63].

A comprehensive view is necessary for robotization, and especially in the implementation of collaborative robots, which allows a definition of an implementation strategy with clearly defined priorities. When implementing robots in the workplace, we therefore considered the definition of safety within the existing legislative measures as a priority. Subsequently, it is possible to focus on other areas related to the solution of technical, economic, ethical or socio-psychological issues.

The presented research had also several limitations. The chosen research method did not allow to take into account the individual specifics of employees when implementing HCI. It was not considered the degree and speed of acceptance of HCI by employees in production and factors that may affect them, such as, e.g., level of knowledge or experience with HCI, skills or age of the employee, etc.

Another limitation of the research was that with the used method of SWOT analysis it was possible to analyse only a limited number of individual criteria, which were divided into four dimensions depending on the external and internal origin and positive or negative impact. At the current state of knowledge, the experts agreed on only 19 criteria included in the SWOT analysis.

The method of SWOT analysis was chosen because the experts involved into the working group, who identified the criteria for the assessment of HCI, are well versed in the problem and are able to use the given method of analysis. Given the expertise in the field and the significant experience of the experts participating in the research, it was possible to compare the criteria, quantify them and determine the appropriate strategy. The repeatable use of SWOT analysis after the implementation of cobots in production requires that sufficient time has elapsed for the effects of the HCI implementation to be felt. The quantification of the criteria makes it possible to transfer them to further research by repeatable use of SWOT analysis. Upon repeatable use of SWOT analysis, some new criteria may arise or disappear when conditions change.

Further direction of research we will focus on which objective and subjective factors influence the degree of HCI acceptance of employees. The objective factors on which we want to focus are the conditions of the working environment in terms of the nature of the production process and environmental working conditions. Analysis of subjective factors makes it possible to compare the results with regard to the employee’s previous experience with working with cobots, intergenerational comparisons, etc. Further follow-up research will also examine the impact of the implementation of cobots and, in general, the introduction of Industry 4.0 on various professions in manufacturing.

## 5. Conclusions

Robots are already the answer to dangerous jobs, such as working with chemical, carcinogenic and toxic substances. The current situation, marked by the widespread transmission of infectious diseases on the scale of an epidemic or even a pandemic, has also affected workers’ perception of their work with cobots. Initial concerns are fading away because of the safety of the “technical colleague” in the transmission and spread of diseases of various kinds. By deploying robots, we can improve workplace ergonomics and also avoid strenuous and repetitive work. So we can say that by putting robots into high-risk operations and reducing the burden of working with loads, we can significantly reduce occupational diseases.

Finally, a need to address a human–robot interface represents an additional challenge. The main task of newly applied intelligent collaboration between human and an industrial robot is to facilitate the repeated, often very demanding and sometimes even dangerous work activity of a human. In today’s industrial manufacturing, in Slovakia, robot and the human workspace is mostly prearranged separately from each other to prevent possible accidents. In the future, however, collaborative workplaces, where the robot will be in close proximity to humans, will be increasingly introduced. Safety and reducing the risk of accidents will remain a priority. In this respect, it is necessary to acknowledge that risks, in this case, cannot be reduced to zero. Injuries will occur, but they will move to other stages of the production process as a part of the adjustment and maintenance processes, when a worker enters the hazardous working or maintenance area of the machine.

## Figures and Tables

**Table 1:** Overview of controls-types, shapes, positions, method of control and control forces (Source: [32]).

Control Type	Shapes, Positions and Frequency of Control	Method of Control	Min. and Max. Forces [N]
**Push Button**	circular, square, rectangular, mushroom-like shape	One finger, palm	min. 2.5/max. 8 min. 2.5/max. 50
**Flip-Flop Switch**	cylindrical, conical, prismatic 2-positioned: min 30° to the sides from the vertical axis, 3-positioned: min. 30° to the sides from the vertical axis and perpendicular to the base	fingers	min. 2.5/max. 10
**Rotary Switch**	circular base, conical grip part, rectangular For visual inspection: max. number of positions 24, min. angle between positions 15° Tactile inspection: max. number of positions 8, min. angle between positions 45°	fingers	min. 2.5/max. 15
**Turn Knob**	cylindrical, conical diameter up to 2.5 cm diameter greater than 2.5 cm	fingers	min. 2.5/max. 4 min. 2.5/max. 15
**Hand Lever**	handle: cylindrical, conic, spherical used permanently, frequently, or rarely	Upper limb Movement of the lever: back and forth to the sides back and forth side up and down (emergency and parking brake) Agricultural and forestry machinery: emergency and parking brake	min. 10/max. 60 min. 10/max. 40 min. 10/max. 120 min. 10/max. 80 min. 10/max. 300 max. 250 max. 295
**Foot Lever**	rectangular, circular, square used continuously, used frequently Agricultural and forestry machinery: clutch pedal accelerator pedal the service and emergency brake pedals other pedals	full foot movement service emergency brake pedal controlled by the movement of the foot in the ankle	min. 10/max. 90 min. 40/max. 400 min. 20/max. 60 max. 245 max. 60 max. 580 max. 150

**Table 2:** SWOT (analysis of Strengths, Weaknesses, Opportunities, and Threats) analysis for the implementation of robotics in industrial settings (Source: own elaboration, 2019).

SWOT Analysis			
Positive			Negative/Harmful
**INTERNAL**			
**STRENGHTS**			**WEAKNESSES**
S1	Elimination of recurring and monotonous work activities	W1	Lack of work experience in the human-robot/cobot system
S2	Elimination of activities in a hazardous work environment	W2	Lack of employee training for new jobs
S3	Reducing and simplifying work with heavy loads	W3	Higher potential for security risks in a collaborative workplace (risk analysis)
S4	Reducing the risk of occupational diseases	W4	Unexamined possible psychosocial burdens
S5	Reducing unilateral physical workload	W5	Safe human-robot/cobot interfaces are not defined
**EXTERNAL**			
	**OPPORTUNITIES**		**THREATS**
O1	Definition of an ergonomically suitable workplace for the human-robot system at the time of design	T1	Elimination of some working positions
O2	Combination of human and robot abilities in a collaborative workplace	T2	Lack of qualified workers
O3	Creation of new jobs	T3	Obsolete legislation in the field of collaborative robots
O4	Responding to the challenges of Industry 4.0 = 4th Industrial Revolution	T4	Fast tightening legislation in terms of OHS
O5	Increasing competitiveness		

**Table 3:** Descriptors according to Saaty’s method (Source: Own elaboration, 2019).

Points	Descriptor
1	Criteria are equally important
3	First criterion is slightly more important than the second one
5	First criterion is fairly more important than the second one
7	First criterion is obviously more important than the second one
9	First criterion is absolutely more important than the second one
2, 4, 6, 8	Slight differences

**Table 4:** Importance of criteria (Source: Own Elaboration, 2019).

Importance of Criteria	Value for Strengths	Value for Weaknesses
Least importance	1	−1
Little importance	2	−2
Average importance	3	−3
Significant importance	4	−4
Strong importance	5	−5

**Table 5:** Pairs preference using Saaty’s method with integrated internal factors analysis (IFAS) matrix (Source: Own elaboration, 2019).

Saaty’s Matrix for Strengths (S) and Weaknesses (W) with Integrated IFAS Matrix											${\left(\prod_{i=1}^{n}x_{i}\right)}^{\frac{1}{n}}$	*v_i_*	Value	Weighted Score
x_i_	S1	S2	S3	S4	S5	W1	W2	W3	W4	W5				
S1	1	1/6	1/3	1/7	1	1/5	3	1/7	5	7	0.69	0.04	1	0.04
S2	6	1	6	1	6	9	9	3	9	9	4.60	0.30	5	1.50
S3	3	1/6	1	1/7	1/3	5	7	1/5	5	7	1.,19	0.08	2	0.16
S4	7	1	7	1	7	6	7	1/3	5	7	3.33	0.22	5	1.09
S5	1	1/6	3	1/7	1	7	5	1/3	7	7	1.45	0.09	2	0.19
W1	5	1/9	1/5	1/6	1/7	1	1/3	1	3	3	0.62	0.04	−1	−0.04
W2	1/3	1/9	1/7	1/7	1/5	3	1	1/4	1/2	1/4	0.33	0.02	−1	−0.02
W3	7	1/3	5	3	3	1	4	1	4	4	2.41	0.16	−4	−0.63
W4	1/5	1/9	1/5	1/5	1/7	1/3	2	1/4	1	1/4	0.30	0.02	−1	−0.02
W5	1/7	1/9	1/7	1/7	1/7	1/3	4	1/4	4	1	0.38	0.02	−1	−0.02
Σ											15.30	1.00		2.25

**Table 6:** Pairs preference using Saaty’s method with integrated external factors analysis (EFAS) matrix (Source: Own elaboration).

Saaty’s Matrix for Opportunities (O) and Threats (T) with Integrated EFAS Matrix										${\left(\prod_{i=1}^{n}x_{i}\right)}^{\frac{1}{n}}$	*v_i_*	Value	Weighted Score
x_i_	O1	O2	O3	O4	O5	T1	T2	T3	T4				
O1	1	1/5	7	7	1/9	5	1/7	5	7	1.44	0.09	2	0.18
O2	5	1	5	7	1/8	5	1/7	7	7	2.09	0.13	3	0.40
O3	1/7	1/5	1	3	1/9	1	1	6	3	0.82	0.05	2	0.10
O4	1/7	1/7	1/3	1	1/9	3	1/8	3	3	0.52	0.03	1	0.03
O5	9	8	9	9	1	9	7	9	9	6.77	0.43	5	2.14
T1	1/5	1/5	1	1/3	1/9	**1**	1/8	1/3	1/5	0.28	0.02	−1	−0.02
T2	7	7	1	8	1/7	8	**1**	8	8	3.13	0.20	−4	−0.79
T3	1/5	1/7	1/6	1/3	1/9	3	1/8	**1**	1/5	0.29	0.02	−1	−0.02
T4	1/7	1/7	1/3	1/3	1/9	5	1/8	5	**1**	0.45	0.03	−1	−0.03
Σ										15.79	1.00		2.00

**Table 7:** Complete SWOT matrix with discrete criteria; Source: Own elaboration.

Complete SWOT Matrix					
**Criteria SI-S5 and W1-W5** **belong to IFAS** **Criteria OI-O5 and T1-T4** **belong to EFAS**		**Strengths (S)**		**Weaknesses (W)**	
		S1	Elimination of reoccurring and monotonous working tasks	W1	Lack of work experience in the human–robot/cobot system
		S2	Removal of activities in a hazardous work environment	W2	Lack of employee training for new jobs positions
		S3	Reducing and simplifying work with heavy loads	W3	Higher potential for security risks in a collaborative workplace (risk analysis)
		S4	Reducing the risk of occupational diseases	W4	Unexamined possible psychosocial burdens
		S5	Reducing unilateral physical workload	W5	Safe human–robot/cobot interfaces are not defined
		4.32		3	
**Opportunities (O)**		**SO Strategy**		**WO Strategy**	
O1	Defining an ergonomically suitable workplace for the human–robot system at the time of design	MAX–MAX strategy of use		MIN–MAX search strategy	
O2	Combination of human and robot abilities in a collaborative workplace	Full use of strengths and opportunities		Maximize opportunities and overcome weaknesses	
O3	Creation of new jobs	4.32 + 3.99 = 8.31		3 + 3.99 = 6.99	
O4	Responding to the challenges of Industry 4.0 = 4th Industrial Revolution				
O5	Increasing competitiveness				
3.99					
**Threats (T)**		**ST Strategy**		**WT Strategy**	
T1	Elimination of some working positions	MAX–MIN strategy of confrontation		MIN–MIN Strategy of avoidance	
T2	Lack of qualified workers	Maximize strengths and suppress threats		Minimize weaknesses while minimizing threats	
T3	Obsolete legislation in the field of collaborative robots	4.32 + 4.14 = 8.47		3 + 4.14 = 7.14	
T4	Fast tightening legislation in terms of OHS				
4.14					

## Data Availability

Not applicable.
